# Fault Diagnosis Method for Excitation Dry-Type Transformer Based on Multi-Channel Vibration Signal and Visual Feature Fusion

**DOI:** 10.3390/s25247460

**Published:** 2025-12-08

**Authors:** Yang Liu, Mingtao Yu, Jingang Wang, Peng Bao, Weiguo Zu, Yinglong Deng, Shiyi Chen, Lijiang Ma, Pengcheng Zhao, Jinyao Dou

**Affiliations:** 1 Baihetan Hydropower Plant, China Yangtze Power Co., Ltd., Liangshan 615400, China; liuyang1226@proton.me (Y.L.); yumt0116@163.com (M.Y.); bpeng331@proton.me (P.B.); weiguo1124@proton.me (W.Z.); dengyinglong@proton.me (Y.D.); chensy0221@proton.me (S.C.); malj1798@proton.me (L.M.); 2 School of Electrical Engineering, Chongqing University, Chongqing 400044, China; jingang@cqu.edu.cn (J.W.); 202311131147t@stu.cqu.edu.cn (J.D.)

**Keywords:** excitation dry-type transformer, multi-channel vibration signal, three-axis feature aggregation, ISDP, ORB, fault diagnosis

## Abstract

To address the limitations of existing fault diagnosis methods for excitation dry-type transformers, such as inadequate utilization of multi-axis vibration data, low recognition accuracy under complex operational conditions, and limited computational efficiency, this paper presents a lightweight fault diagnosis approach based on the fusion of multi-channel vibration signals and visual features. Initially, a multi-physics field coupling simulation model of the excitation dry-type transformer is developed. Vibration data collected from field-installed three-axis sensors are combined to generate typical fault samples, including normal operation, winding looseness, core looseness, and winding eccentricity. Due to the high dimensionality of vibration signals, the Symmetrized Dot Pattern (ISDP) method is extended to aggregate and map time- and frequency-domain information from the x-, y-, and z-axes into a two-dimensional feature map. To optimize the inter-class separability and intra-class consistency of the map, Particle Swarm Optimization (PSO) is employed to adaptively adjust the angle gain factor (η) and time delay coefficient (t). Keypoint descriptors are then extracted from the map using the Oriented FAST and Rotated BRIEF (ORB) feature extraction operator, which improves computational efficiency while maintaining sensitivity to local details. Finally, an efficient fault classification model is constructed using an Adaptive Boosting Support Vector Machine (Adaboost-SVM) to achieve robust fault mode recognition across multiple operating conditions. Experimental results demonstrate that the proposed method achieves a fault diagnosis accuracy of 94.00%, outperforming signal-to-image techniques such as Gramian Angular Field (GAF), Recurrence Plot (RP), and Markov Transition Field (MTF), as well as deep learning models based on Convolutional Neural Networks (CNN) in both training and testing time. Additionally, the method exhibits superior stability and robustness in repeated trials. This approach is well-suited for online monitoring and rapid diagnosis in resource-constrained environments, offering significant engineering value in enhancing the operational safety and reliability of excitation dry-type transformers.

## 1. Introduction

With the continuous expansion of power systems and the advancement of intelligent technologies, the operational safety and reliability of transformers have attracted growing attention. Dry-type transformers, valued for their inherent safety, environmental compatibility, and maintenance-free operation, are widely deployed in applications such as railway transportation, clean-energy power plants, and distributed generation systems [1]. Among them, excitation dry-type transformers play a critical role in supplying stable excitation currents for hydropower station excitation systems, typically operating under low-load or no-load conditions. Their operational stability is directly associated with maintaining voltage balance and magnetic-circuit continuity. However, when subjected to dynamic loads from synchronous motor excitation or power electronic converters, the core and windings undergo fluctuating electromagnetic forces, leading to pronounced mechanical vibrations [2,3,4]. Over time, these vibrations may give rise to mechanical defects such as core loosening and winding loosening, which disrupt magnetic flux distribution, degrade electrical performance, and potentially cause insulation breakdown and partial discharge. Consequently, elucidating the vibration mechanisms of excitation dry-type transformers and developing efficient diagnostic methods for mechanical fault detection are essential for improving equipment efficiency, prolonging service life, and ensuring the safe and reliable operation of modern power systems.

In recent years, transformer fault diagnosis has transitioned from manual, experience-based methods to intelligent models that integrate multiple disciplines, including electrical detection, oil analysis, vibration monitoring, and advanced algorithms. These methods differ significantly in diagnostic accuracy, real-time performance, and applicability, each offering distinct advantages and limitations. Table 1 provides a comparison of key diagnostic methods, summarizing their core principles, strengths, weaknesses, and application fields.

As shown in Table 1, existing diagnostic methods face challenges in balancing real-time performance with diagnostic accuracy. Single-signal methods are limited by inadequate feature representation, while multi-source fusion methods are constrained by complexity and high costs. For excitation dry-type transformers, their sensitivity to faults through vibration signals is affected by interference from factors such as grid harmonics and load fluctuations, making it difficult to capture weak fault characteristics.

Vibration signals provide rich information about the mechanical and electromagnetic characteristics of transformers, enabling non-intrusive, real-time monitoring. When their multi-dimensional features are effectively integrated, they can overcome the limitations of existing methods. Early work by Tang et al. [14] compared vibration frequency-domain data from normal and faulty transformers but struggled to capture weak fault characteristics. Later, Yu et al. [15] applied FFT to vibration signals and used a BP neural network for fault diagnosis. However, the BP network’s slow convergence and instability hindered its accuracy. Wu et al. [16] proposed an online vibration frequency response analysis (VFRA) method, successfully identifying winding loosening faults but failing to account for capacitive effects and harmonic coupling under high-frequency conditions. More recently, deep learning methods have been applied to 2D image classification of vibration signals. Xiao et al. [17] used the Granian Angle Summation Field (GASF) for fault diagnosis, but this method captures only one-dimensional features, missing important three-dimensional and time-frequency coupling information. Hong et al. [18] converted vibration data with load information into images and used a CNN for classification, achieving fault diagnosis. However, the model’s trade-off between training time and accuracy makes it unsuitable for real-time applications. Overall, current research often fails to fully exploit the spatiotemporal structure of three-axis vibration signals, limiting both diagnostic accuracy and computational efficiency for real-time applications.

To address the aforementioned challenges, this study proposes a lightweight fault diagnosis method for excitation dry-type transformers that integrates multi-channel vibration signals with visual feature fusion, emphasizing operational characteristic aggregation and efficient feature extraction. The main innovations of the proposed approach are summarized as follows:

- Three-Axis Feature Aggregation and Dimensionality Reduction: An Improved Symmetrized Dot Pattern (ISDP) method is developed to aggregate and project the time- and frequency-domain features of x-, y-, and z-axis vibration signals into a unified two-dimensional map. This strategy preserves feature integrity while reducing data dimensionality and improving computational efficiency.
- Self-Adaptive Parameter Optimization: Particle Swarm Optimization (PSO) is employed to automatically adjust the angle gain factor (*η*) and time-delay coefficient (*t*) of the ISDP method. This adaptive mechanism enhances inter-class separability and intra-class compactness of the feature map, effectively mitigating feature loss caused by manual, experience-based parameter selection.
- Efficient Local Feature Extraction: The Oriented FAST and Rotated BRIEF (ORB) operator is applied to extract keypoint descriptors from the generated feature maps. This enables accurate capture of local distortions and texture variations while substantially reducing computational cost.
- Lightweight Classification Framework: An Adaptive Boosting Support Vector Machine (Adaboost-SVM) is integrated as the classification module, striking a balance between diagnostic accuracy and computational efficiency. This framework is particularly advantageous for small-sample scenarios and real-time diagnostic requirements in practical engineering environments.

## 2. Vibration Modeling and Simulation of Excitation Dry-Type Transformer

### 2.1. Vibration Mechanisms

Vibrations in excited dry-type transformers arise from two main mechanisms: mechanical stress in the core due to the magnetostrictive effect and the Lorentz force acting on the windings in the magnetic field [19]. These forces are transmitted to the transformer components, triggering a structural response. Under normal conditions, vibration acceleration in the core is proportional to the square of the applied voltage, with the vibration frequency typically occurring at twice the excitation voltage frequency. For a 50 Hz power supply, core vibrations primarily occur at 100 Hz, and the axial and radial electromagnetic forces in the winding also exhibit frequencies at 100 Hz.

### 2.2. Multiphysics Simulation Model

This paper investigates the ZLDCB-3200/24/$\sqrt{3}$/1.1 resin-insulated dry-type transformer, supplied by Sunten Electric Equipment Co., Ltd. (Wuhan, China), using a 3D model developed with COMSOL Multiphysics 6.1 to analyze the vibration signal characteristics under various operating conditions. To balance accuracy and computational efficiency, structural simplifications were made [20,21], excluding minor components such as tap switches and clamps, while retaining the epoxy resin encapsulation around the windings. Non-ferromagnetic elements, including inter-turn insulation and phase-to-phase insulation, were omitted. Key parameters are summarized in Table 2.

Simulation models are developed to analyze the vibration response of the core and winding under typical structural faults, such as winding loosening, core loosening, and winding eccentricity. Winding pin bolt loosening is simulated by adjusting the preload force applied by the spacers, which is related to changes in the winding material’s Young’s modulus. Similarly, core loosening is modeled by reducing its elastic modulus [22,23]. The corresponding mechanical parameters for different degrees of loosening are summarized in Table 3.

**Figure 1:**
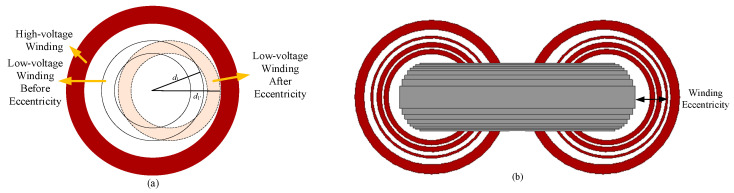
Winding eccentricity fault simulation setup in an excitation dry-type transformer. (**a**) Top view model of winding eccentricity fault; (**b**) 3D simulation model of winding eccentricity fault.

The specific fault simulation configuration for winding eccentricity is illustrated in Figure 1.

### 2.3. Parametric Scanning and Calibration of the Model

Excitation dry-type transformers typically operate under normal conditions, with fault conditions being rare. Simulating winding eccentricity faults is challenging due to their destructive nature and safety risks, limiting fault-condition data for research. To address this, the study simulates winding eccentricity faults under different load conditions using a multi-physics model. This compensates for the lack of real fault samples and broadens data coverage. COMSOL Multiphysics is used to optimize key model parameters (e.g., load resistance, winding DC resistance) to minimize simulation errors and improve data reliability [24], with settings summarized in Table 4.

## 3. Fault Diagnosis Methodology

### 3.1. SDP Method (Symmetrized Dot Pattern Method)

The Symmetrized Dot Pattern (SDP) method converts one-dimensional time-series signals into two-dimensional polar coordinate maps, revealing underlying nonlinear characteristics and dynamic behaviors [25]. By transforming both time-domain and frequency-domain information into a point distribution pattern, the SDP image captures both aspects. Shape features, such as thickness and curvature, represent different operating states. The formula for mapping the signal to polar coordinates is provided in the following equations.


(1) $$r(i)=\frac{z_{i}-z_{\min}}{z_{\max}-z_{\min}}$$

(2) $$\omega_{a}=\frac{360m}{n}$$

(3) $$\delta (i)=\omega_{a}+\frac{z_{i+t}-z_{\min}}{z_{\max}-z_{\min}}\eta$$

(4) $$\lambda (i)=\omega_{a}-\frac{z_{i+t}-z_{\min}}{z_{\max}-z_{\min}}\eta$$

**Figure 2:**
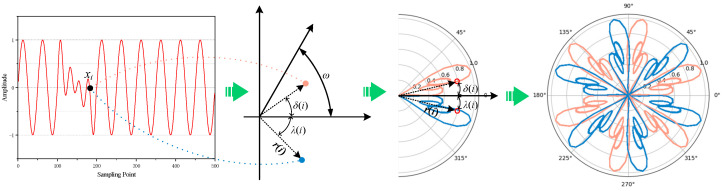
Principle of the SDP method. The color “light coral” represents the counterclockwise rotation angle of the mirror symmetry plane, while the color “blue” represents the clockwise rotation angle of the mirror symmetry plane.

In the formula, *r*(*i*) represents the polar radius of the *i*-th vibration signal, *z_i_* is the amplitude of the *i*-th vibration signal, *z*_max_ is the maximum amplitude in the one-dimensional vibration sequence, and *z*_min_ is the minimum amplitude in the one-dimensional vibration sequence. *t* denotes the delay offset, *η* is the angle gain factor, *ω_a_* is the angle of the mirror symmetry axis, and *m* = [0, 1, …, *n* − 1], where *n* represents the number of mirror symmetry planes. To achieve clear symmetry in the pattern, *n* is typically set to 6. *δ*(*i*) is the counterclockwise rotation angle of the mirror symmetry plane, and *λ*(*i*) is the clockwise rotation angle of the mirror symmetry plane. The conversion principle is illustrated in Figure 2.

### 3.2. ISDP Method (Improved Symmetrized Dot Pattern Method)

The traditional SDP method transforms single-axis vibration signals into 2D maps for fault visualization. However, excitation dry-type transformers inherently produce 3D vibration signals, with distinct fault signatures on the x-, y-, and z-axes. Relying on a single-axis signal can overlook critical features and increase susceptibility to noise, reducing diagnostic accuracy and stability.

By integrating information from all three axes, a more comprehensive feature representation is achieved, enhancing fault recognition and robustness under complex conditions. However, creating separate SDP maps for each axis increases preprocessing complexity, dimensionality, and computational demand. This study addresses these challenges by merging the 2D maps from the x-, y-, and z-axes through image stitching, which preserves distinctive features while improving efficiency.

Taking the three-axis vibration signal time-series matrix **V** as an example, **V** can be expressed as in (3).


(5) $$V=\left[\begin{array}{ccc} v_{x1} & v_{y1} & v_{z1}\\ v_{x2} & v_{y2} & v_{z2}\\ \vdots & \vdots & \vdots \\ v_{xn} & v_{yn} & v_{zn} \end{array}\right]$$

In the equation, *v_x_*, *v_y,_* and *v_z_* denote the time-domain vibration signals along the x-, y-, and z-axes, respectively, while *n* represents the length of the vibration signal. The three-axis vibration data matrix **V** can then be mapped to polar coordinates using (4)–(6).


(6) $$r(i,j)=\frac{V_{ij}-V_{j\min}}{V_{j\max}-V_{j\min}}$$

(7) $$\delta (i,j)=\omega_{a}+\frac{V_{(i+t)j}-V_{\min}}{V_{\max}-V_{\min}}\eta$$

(8) $$\lambda (i,j)=\omega_{a}-\frac{V_{(i+t)j}-V_{\min}}{V_{\max}-V_{\min}}\eta$$

**Figure 3:**
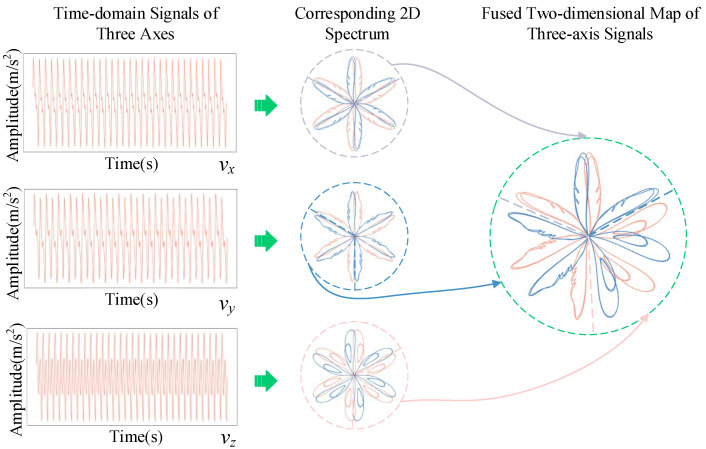
Principle of ISDP method. The arrows indicate the regions where *v_x_*, *v_y_*, and *v_z_* are extracted and stitched together from the original figure.

In the equation, **V***_ij_* represents the *i*-th sampling point of the vibration signal on the *j*-axis, where *j* = [1, 2, 3]. **V***_j_*_max_ is the maximum amplitude of the *j*-th axis in matrix **V**, and **V***_j_*_min_ is the minimum amplitude of the *j* axis in matrix **V**. *δ*(*i, j*) is the counterclockwise rotation angle of the mirror-symmetrical plane of the *j*-th column data in matrix **V**, and *λ*(*i, j*) is the clockwise rotation angle of the mirror-symmetrical plane of the *j*-th column data in matrix **V**. In the improved SDP method, the mirror-symmetrical axis angle is *ω_a_* = 60 * (2*j* − *k*), where *k*∈{0, 1}. The principle of the improved SDP image transformation is shown in Figure 3.

### 3.3. ORB Keypoint Feature Description Method

**Figure 4:**
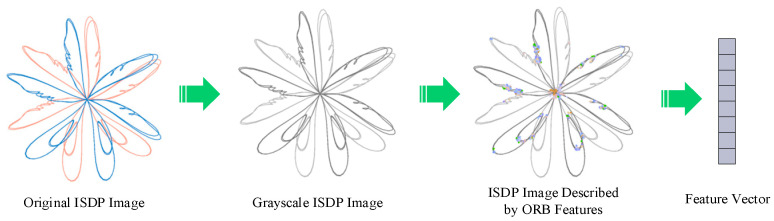
ORB descriptor algorithm extraction process.

In this study, the Oriented FAST and Rotated BRIEF (ORB) algorithm is employed to extract features from the ISDP images generated by the core vibration signals of the excitation dry-type transformer. The overall processing workflow is illustrated in Figure 4 [26]. First, the ISDP method converts the three-axis vibration signals into a two-dimensional feature map, enabling visualization of their time–frequency characteristics. The ISDP map is then converted to grayscale, which reduces computational complexity while preserving essential structural information. Subsequently, the ORB algorithm is applied to detect local keypoints and compute descriptors from the grayscale image. To improve keypoint detection effectiveness, an edge-region priority mechanism is introduced by applying a keypoint mask, guiding the ORB algorithm to prioritize feature extraction from edge regions and thereby mitigating the loss of discriminative features in smooth or redundant interior areas [27]. Finally, the extracted ORB descriptors—binary vectors of 256 bits (32 bytes)—are clustered into N classes using the K-Means algorithm, with keypoints from different classes marked in distinct colors to enhance visualization and feature discrimination.

**Figure 5:**
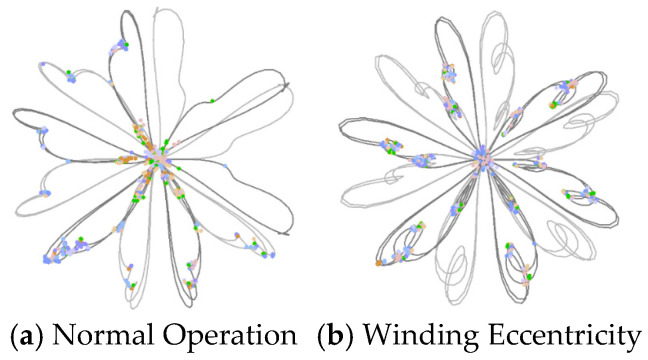
ORB keypoint distribution of feature maps under normal operation and winding eccentricity conditions.

Using normal operating samples and winding eccentricity samples as examples, feature extraction was performed with the ORB algorithm, and the resulting keypoint distributions are shown in Figure 5. Under normal operating conditions, the extracted keypoints are evenly distributed, primarily concentrated along the “petal” edges and symmetric structural regions, indicating the regularity and stability of the vibration signal map. In contrast, under winding eccentricity conditions, the keypoints cluster in areas with significant deformation, revealing asymmetry and dense grouping. These patterns effectively highlight the abnormal vibration caused by mechanical eccentricity. This demonstrates the ORB algorithm’s robustness and sensitivity in capturing local image features under different operating states of the transformer.

### 3.4. Construction of the Adaboost-SVM Fault Diagnosis Model

To meet the demands of real-time and rapid fault diagnosis in excitation transformers, we employ the Support Vector Machine (SVM) as the base classifier within the Adaboost framework [28,29,30].

Given a dataset containing *n* samples, K = {(*x*_1_, *y*_1_), …, (*x_n_*, *y_n_*)}, where *x_n_* represents the feature vector of the *n*-th sample and *y_n_* is its corresponding label, taking values of −1 or 1, the initial weight *α_i_* for each sample is assigned an equal value and initialized as (9).


(9) $$\alpha_{i}=\frac{1}{n},\;i=1,2,\ldots ,n$$

In the *t*-th iteration, a weighted sample set is used to train the SVM classifier. The SVM with a linear kernel performs well on high-dimensional vibration signal feature data and exhibits relatively high computational efficiency. The weak classifier *h_t_*(*x*) obtained after training is expressed as (10).


(10) $$y_{t}^{*}=R(x_{n},y_{n},\alpha_{n})$$

In the formula, *R* represents the output of the SVM classifier after being applied to the weighted sample set; *a_n_* denotes the weight of the sample; and *y_n_* represents the corresponding label.

After obtaining the weak classifier $y_{t}^{*}$, the misclassified samples in the weighted sample set are re-weighted, and the classification error *ϵ_t_* is calculated according to (11).


(11) $$\epsilon_{t}=\sum_{i=1}^{n}\alpha_{i}\cdot I\left(y_{i}\neq h_{t}\left(x_{i}\right)\right)$$

In the formula, $I(y_{i}\neq y_{t}^{*})$ represents an indicator function. When sample *I* is misclassified, its value is 1; otherwise, it is 0. The classification error *ϵ_t_* is used to measure the performance of the weak classifier. A smaller error indicates better classification performance.

Based on the classification error *ϵ_t_*, the sample weights are updated according to the update rule shown in (12).


(12) $$\alpha_{i+1}=\alpha_{i}\cdot \exp\left(-\eta_{t}\cdot y_{i}\cdot h_{t}(x_{i})\right)$$

In the formula, *η_t_* represents the adjustment coefficient.

After updating the sample weights, they are normalized according to Equation (13) to ensure that the total weight of all samples equals 1. This normalization prevents any single sample from exerting excessive influence during the training process, thereby ensuring the stability of the model.


(13) $$\alpha_{i+1}=\frac{\alpha_{i}}{\sum_{i=1}^{n}\alpha_{i}}$$

After *m* rounds of iteration, the final strong classifier *H*(*x*) is obtained by the weighted combination of all weak classifiers *h_t_*(*x*), as shown in (14).


(14) $$H(x)=\mathrm{sign}\left(\sum_{t=1}^{m}\eta_{t}h_{t}(x)\right)$$

In the formula, the sign function outputs the final classification result based on the weighted sum of all weak classifiers. The contribution of each weak classifier is determined by its error *η_t_*, with classifiers having smaller errors contributing more.

### 3.5. Diagnostic Process

**Figure 6:**
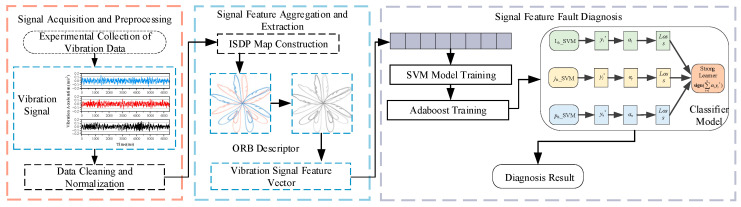
Fault diagnosis framework for excitation dry-type transformers based on multi-channel vibration signal visual features.

Based on the above methodology, this study develops a fault diagnosis framework for excitation dry-type transformers that leverages multi-channel vibration signal visual features. The framework comprises three core stages: (i) vibration signal acquisition and preprocessing; (ii) multi-channel visual feature extraction and fusion; and (iii) fault mode classification and discrimination. The complete diagnostic process, illustrated in Figure 6, achieves end-to-end processing and recognition, spanning from raw vibration data acquisition to final fault category output.

In the signal sampling and preprocessing stage, raw vibration signals are segmented using a sliding window strategy, followed by normalization and denoising to generate stable inputs for feature extraction. In the aggregation and extraction stage, high-frequency three-axis vibration signals are fused and visualized using the improved ISDP method, creating feature maps for the x-, y-, and z-axes under various conditions. The ORB algorithm encodes local details, extracts keypoint descriptors, and converts them into vectorized vibration features. In the fault diagnosis stage, the Adaboost model with SVM weak classifiers is trained on labeled samples to build a discriminative model. In field applications, a pseudo-label self-training mechanism updates the model with real-time unlabeled data, enhancing its adaptability to long-term operational changes.

## 4. Field Experiments and Dataset Construction

### 4.1. Selection of Measurement Point Locations

**Figure 7:**
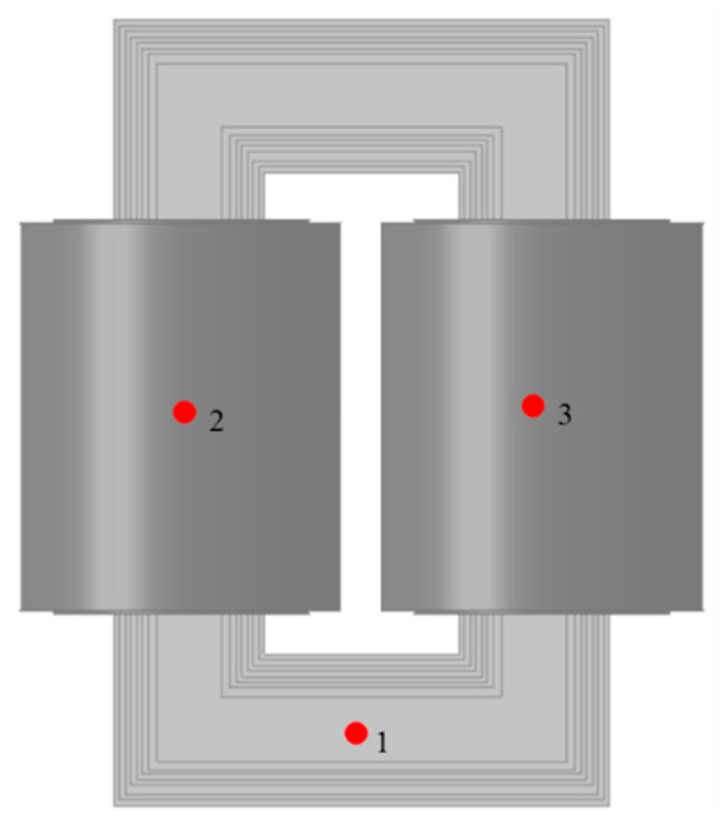
Vibration signal measurement point locations of the excitation dry-type transformer.

The selection of measurement points balances the need for accurate signal acquisition with considerations of structural characteristics, safety, and wiring feasibility [31]. Three representative locations were chosen for vibration signal collection: the bottom of the winding near the core, and the midpoints of the left and right column windings (see Figure 7).

**Figure 8:**
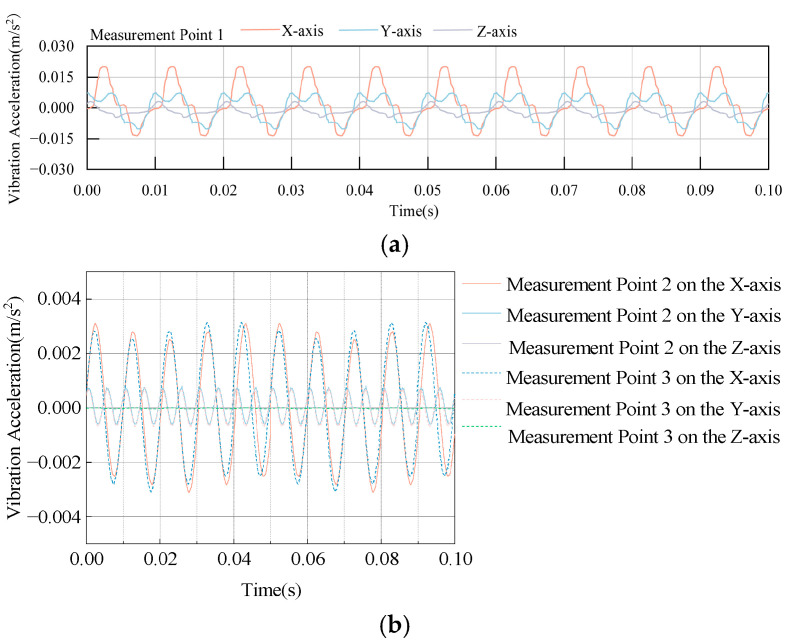
Acceleration curves at different measurement point locations. (**a**) Core acceleration curve at measurement point 1; (**b**) Winding acceleration curves at measurement points 2–3.

Under normal operating conditions at the transformer’s rated load, acceleration time-domain signals were collected from each measurement point to analyze their response characteristics. Figure 8 displays the acceleration response curves of the core and windings at the three measurement points over 10 excitation cycles, highlighting distinct vibration characteristics at different structural positions.

As shown in Figure 8, the core measurement point (Point 1) exhibits higher vibration acceleration than the winding points (Points 2 and 3), primarily due to differences in structural stiffness. The windings, encapsulated in epoxy resin, are less responsive to vibrations, while the core, secured by clamping components, is more susceptible to pronounced vibrations. Therefore, the core measurement point was selected for monitoring to capture a signal with distinct characteristics.

### 4.2. On-Site Data Collection

**Figure 9:**
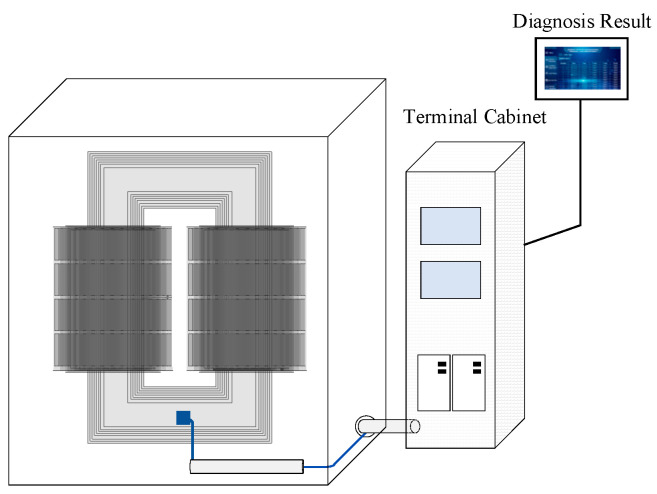
Vibration sensor installation scheme.

To enable real-time monitoring and fault identification in excitation dry-type transformers, three-axis vibration sensors are mounted on the transformer body. Using phase A as an example, the sensor is positioned at the midpoint of the core (Figure 9) due to its sensitivity to electromagnetic excitation, capturing structural vibrations induced by electromagnetic forces. A high-sensitivity three-axis accelerometer records vibrations along the x-, y-, and z-axes [32], with a sampling frequency sufficient to detect both power frequency (50 Hz) and higher-order harmonics (e.g., 100 Hz, 150 Hz). The sensor output is transmitted via RS485 to the data acquisition system [33], ensuring reliable long-distance transmission and resistance to interference.

**Figure 10:**
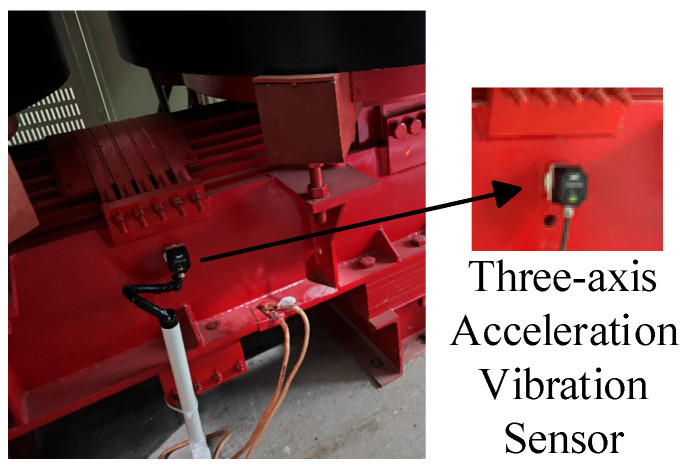
Field installation location of vibration sensors.

Electromagnetic shielding and mechanical reinforcement were applied to the sensor wiring to minimize interference and enhance data accuracy [34]. The system displays real-time sensor outputs, including time-domain waveforms and spectrograms, and also provides fault diagnosis results. This approach improves the system’s robustness in complex power environments, ensuring stable data for accurate fault identification. The field layout is shown in Figure 10.

**Figure 11:**
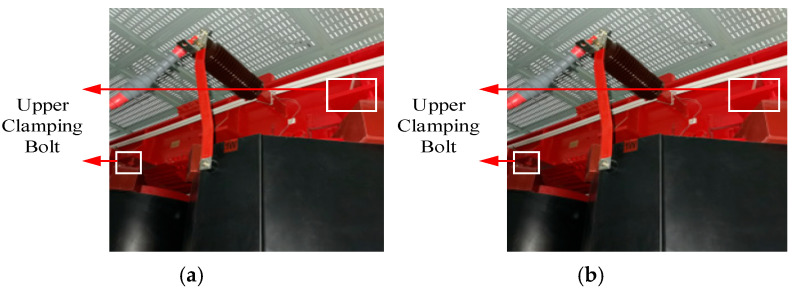
Field fault simulation setup for an excitation dry-type transformer. (**a**) Winding loosening fault simulation setup; (**b**) Core loosening fault simulation setup.

Field data were collected under three operating conditions: (i) winding loosening fault, (ii) core loosening fault, and (iii) normal operation under varying loads and voltages. Faults were simulated by loosening the winding clamping bolts and the core fastening bolts. The field fault configurations are shown in Figure 11.

### 4.3. Dataset Construction

To validate the proposed fault diagnosis method, a comprehensive dataset was created by combining on-site vibration signals with multi-physics simulation data, covering various transformer failure modes and operating conditions for model training and validation.

A three-axis vibration sensor was installed on the excitation dry-type transformer to collect on-site data, including normal operation under varying loads and typical fault conditions such as winding loosening, core loosening, and winding eccentricity. Due to the difficulty of obtaining winding eccentricity samples on-site, a multi-physics simulation model using COMSOL Multiphysics was used to generate supplementary data. The dataset was divided into training, test, and validation sets with a 60–20–20% ratio (Table 5).

To improve model accuracy, all collected vibration signals were preprocessed, including normalization to standardize fault characteristics and wavelet denoising to reduce high-frequency noise and environmental interference.

## 5. Analysis of the Experimental Results

### 5.1. Evaluation Metric

To more intuitively demonstrate the recognition ability of each model across different fault categories, five widely used classification performance metrics—accuracy (Acc), precision (Pre), specificity (Spe), recall (Rec), and F1 score (F1)—are computed separately for each fault category. For each category, all other fault categories are combined into a single group. The calculation formulas for Pre, Spe, Rec, and F1 are provided in Equations (15)–(18).


(15) $$\mathrm{Pre}=\frac{TP}{TP+FP}$$

(16) $$\mathrm{Spe}=\frac{TN}{TN+FP}$$

(17) $$\mathrm{Rec}=\frac{TP}{TP+FN}$$

(18) $$F1=\frac{2\times TP}{2\times TP+FP+FN}$$

In the formula, *TP* (True Positive) refers to correctly identified positive samples, where the model successfully predicts true positive samples as the positive class, indicating that faults are correctly detected. *TN* (True Negative) refers to correctly identified negative samples, where the model correctly classifies true negative samples as the negative class, indicating accurate recognition of fault-free situations. *FP* (False Positive) refers to incorrect positive predictions, where the model mistakenly classifies true negative samples as positive, resulting in a “false alarm” where normal equipment is misidentified as faulty. *FN* (False Negative) refers to incorrect negative predictions, where the model mistakenly classifies true positive samples as negative, leading to “missed detection,” where faulty equipment is not identified.

The overall classification performance of the model is evaluated using four indicators: macro-average precision $\bar{\mathrm{Pr}e}$, macro-average F1 score $\bar{F1}$, average training time (*t_d_*), and average test time (*t_s_*). The calculation methods for macro-average precision and macro-average F1 score are provided in Equations (19) and (20), respectively.


(19) $$\bar{\mathrm{Pr}e}=\frac{1}{N}\sum_{i=1}^{N}{\mathrm{Pre}}_{i}$$

(20) $$\bar{F1}=\frac{1}{N}\sum_{i=1}^{N}{F1\text{-}\mathrm{Score}}_{i}$$

### 5.2. Simulation Results of Vibration Signals

**Figure 12:**
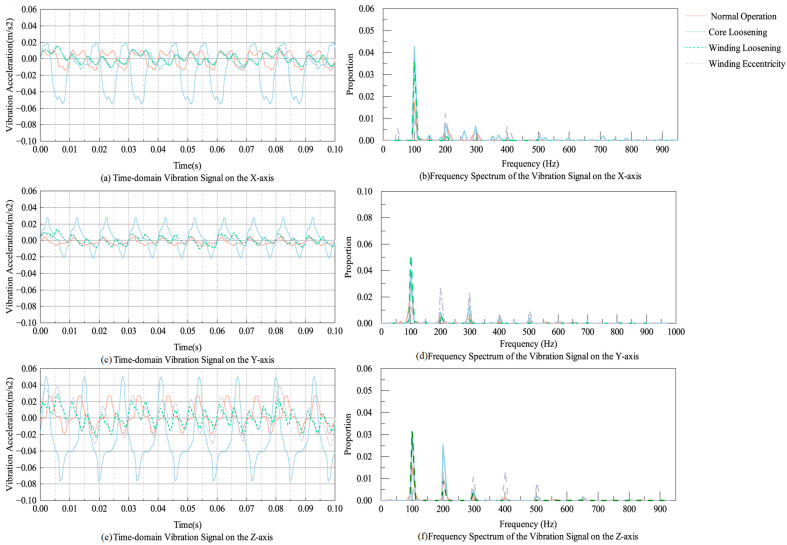
Simulation results of vibration signals under different operating conditions. (**a**) Time-domain vibration signal on the X-axis; (**b**) frequency spectrum of the vibration signal on the X-axis; (**c**) time-domain vibration signal on the Y-axis; (**d**) frequency spectrum of the vibration signal on the Y-axis; (**e**) time-domain vibration signal on the Z-axis; (**f**) frequency spectrum of the vibration signal on the Z-axis.

The simulation results revealed that the vibration acceleration amplitude at each measurement point on the core in the z-axis direction was higher than that in the x- and y-axes, indicating that the primary deformation of the core occurred along the z-axis. In addition to the 100 Hz fundamental frequency, the vibration signals also contain harmonic components due to stress-dependent variations in Young’s modulus, imparting nonlinear characteristics to the system [35]. To investigate the vibration response under fault conditions, a right column winding fault was selected as a representative case. The time–frequency response of the acceleration at measurement point 1 under different operating conditions is shown in Figure 12.

As shown in Figure 12, the vibration acceleration exhibits distinct time–frequency characteristics under various conditions. During normal operation, the vibration signals are dominated by a 100 Hz fundamental frequency, with additional high-frequency components. Under core loosening, the vibration amplitude increases to 0.049 m/s^2^, indicating heightened vibration energy. In the case of winding loosening, the relative energy contribution of the 100 Hz frequency increases, while for winding eccentricity, high-frequency harmonics emerge, highlighting structural asymmetry. Overall, the time–frequency features demonstrate the method’s sensitivity to different structural faults, validating its effectiveness in distinguishing transformer operating states.

### 5.3. Simulation Results of Fault Simulations

To validate the simulation model under different operating conditions, this study compares the similarity between simulated and measured data for both no-load and load conditions. For the no-load case, three operating states—normal operation, core loosening, and winding loosening—are examined. For the load case, simulation data are compared with field measurements at a 75% load rate. Similarity is evaluated in both the time and frequency domains: spectral coherence is used for frequency-domain similarity, and Dynamic Time Warping (DTW) distance is used for time-domain similarity [36]. The results are summarized in Table 6.

As shown in Table 6, after parameter optimization, the relative error between the simulation and measured data is within 5%. The DTW distance remains below 0.4, indicating that the time-domain characteristics of the simulated and field data align closely, with minimal temporal shifts or distortions. This is crucial for the model’s ability to generalize to real-world data. Additionally, spectral coherence exceeds 0.7, confirming that the frequency-domain patterns of both datasets are consistent, ensuring proper alignment of vibrational frequencies and harmonic components. These results validate the accuracy and reliability of the simulation model. Consequently, the parameter-optimized simulation method is used to generate winding eccentricity fault samples, which supplement the training dataset and enhance the model’s generalization.

### 5.4. Analysis of Fault Diagnosis Results

#### 5.4.1. Parameter Optimization Using Particle Swarm Optimization (PSO)

In existing studies, the time-delay coefficient t in the SDP method is typically set empirically within the range of 0–10, while the angle gain factor *η* is restricted to 0–60°, making it difficult to achieve optimal parameter selection [37]. This often results in information loss during feature construction. To address this issue, this paper introduces the Particle Swarm Optimization (PSO) algorithm to jointly optimize the ISDP parameters, namely the angle gain factor *η* and the time-delay coefficient *t*. The PSO configuration is as follows: particle population size of 25, learning factors *a*_1_ = *a*_2_ = 1.2, inertia weight *ω* = 0.6, and 120 iterations. The search ranges are defined as [10°, 60°] for the angle gain factor *η* and [1, 10] for the time-delay coefficient *t*. The fitness function is defined by the quality of feature extraction after map construction, which guides the optimization process.

#### 5.4.2. ISDP Image Similarity and Feature Extraction

To ensure the significance of image features across different fault types after ISDP image conversion, the fitness function is defined as the average similarity between the ISDP images. The digital matrix *F* corresponding to the ISDP image is represented by Equation (21).


(21) $$F=\left[\begin{array}{cccc} p(0,0) & p(0,1) & \ldots & p(0,n-1)\\ p(1,0) & p(1,1) & \ldots & p(1,n-1)\\ \vdots & \vdots & \ddots & \vdots \\ p(m-1,0) & p(m-1,1) & \ldots & p(m-1,n-1) \end{array}\right]$$

In the formula, *p*(*x, y*) represents the pixel value at the coordinate (*x, y*), *m* is the number of pixel columns, and *n* is the number of pixel rows. The similarity *R*(**M**, **N**) between the pixel matrices *M* and *N* of two ISDP images under different fault conditions can be expressed by (22).


(22) $$R(M,N)=\frac{\sum_{m}\sum_{n}\left(M_{mn}-\bar{M})(N_{mn}-\bar{N}\right)}{\sqrt{\left[\sum_{m}\sum_{n}{\left(M_{mn}-\bar{M}\right)}^{2}\right]\left[\sum_{m}\sum_{n}{\left(N_{mn}-\bar{N}\right)}^{2}\right]}}$$

In the formula, $\bar{M}$ represents the average value of the **M** pixels in the pixel matrix, and $\bar{N}$ represents the average value of the **N** pixels in the pixel matrix. Therefore, the similarity matrix *R* of ISDP images across different fault types can be obtained, as shown in (23).


(23) $$R=(R_{1,2}(M_{1},N_{2}),R_{1,3}(M_{1},N_{3}),R_{1,4}(M_{1},N_{4})\cdots ,R_{i-1,i}(M_{i-1},N_{i}))$$

In the formula, *i* represents the number of fault types in dry-type transformers.

Ultimately, the fitness function *Fit* is defined as the average similarity, as shown in Equation (24).


(24) $$Fit=\bar{R}=\frac{\sum_{j=1}^{i-1}\sum_{k=j+1}^{i}R_{j,k}(M_{j},N_{k})}{(i-1)\times i/2}$$

**Figure 13:**
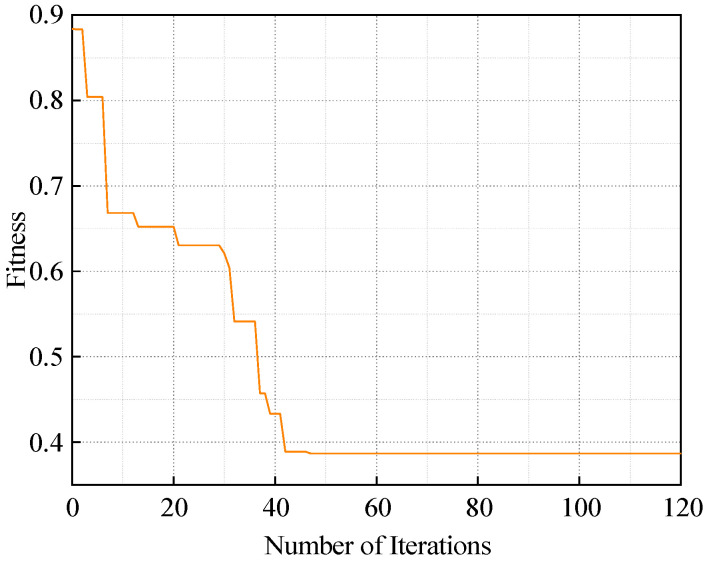
Particle swarm optimization algorithm iteration curve.

The final optimization results are illustrated in Figure 13.

#### 5.4.3. ISDP Feature Map Generation and Analysis

**Figure 14:**
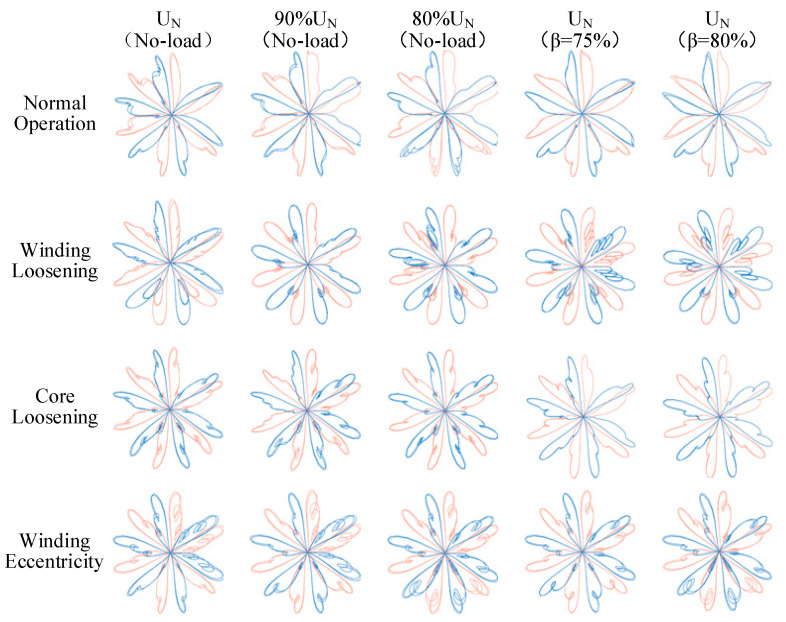
Vibration signal feature aggregation and extraction effect diagram.

Using the ISDP parameters optimized by the Particle Swarm Optimization algorithm, the collected vibration signal samples were aggregated and transformed to generate the corresponding ISDP images. Figure 14 presents the ISDP feature maps obtained after three-axis fusion of vibration signals under five representative operating conditions: no-load rated voltage, no-load 90% rated voltage, no-load 80% rated voltage, 75% load rate, and 80% load rate. As shown, the ISDP images corresponding to different fault types exhibit pronounced differences in overall petal shape, edge contours, and texture distribution, demonstrating strong discriminability. Conversely, for the same fault type under different operating conditions, the structural features remain highly consistent, indicating that the method achieves high intra-class stability while maintaining strong inter-class separability.

**Figure 15:**
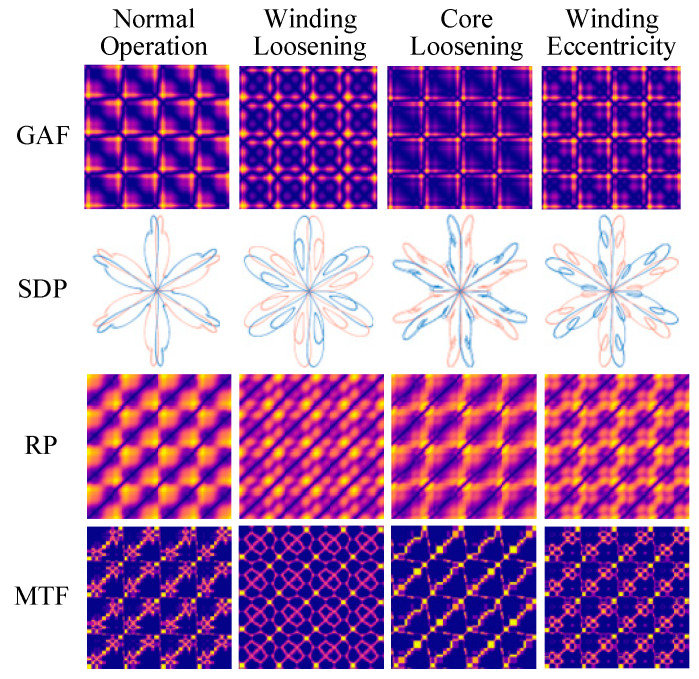
Reproduction results of other signal-to-image transformation methods.

To validate the effectiveness of the ISDP method for vibration signal feature aggregation and extraction, this study compares it with several commonly used one-dimensional to two-dimensional signal transformation techniques. The selected methods include the traditional Symmetrized Dot Pattern (SDP), Gramian Angular Fields (GAFs), Markov Transition Field (MTF), Recurrence Plot (RP), and the improved SDP (ISDP) proposed in this paper [38]. Figure 15 illustrates the image results obtained by processing a set of z-axis vibration signals from the excitation dry-type transformer under four operating states—normal operation, winding loosening, core loosening, and winding eccentricity—during no-load rated voltage operation.

#### 5.4.4. Adaboost-SVM Parameter Optimization for Fault Diagnosis

**Figure 16:**
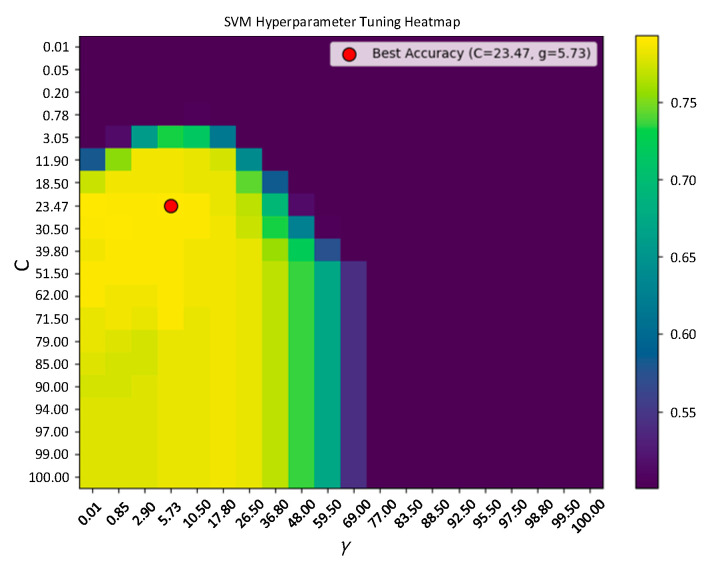
Grid search results for SVM parameters.

Before training the Adaboost algorithm, the key parameters of the SVM classifier, namely the penalty factor *C* and the kernel function parameter *γ*, are optimized using grid search. The search range for *C* and *γ* was set to [10^−1^, 10^2^], and the optimal values were *C* = 23.47, *γ* = 5.73, resulting in a classification accuracy of 77.31% (Figure 16).

**Figure 17:**
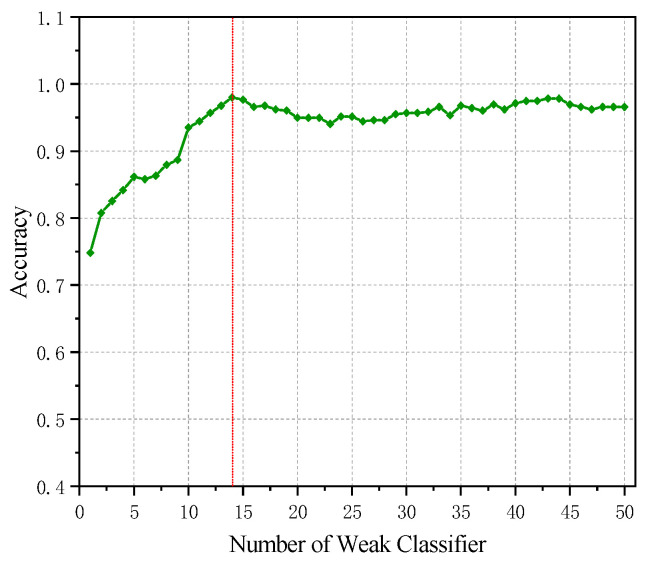
Optimization curve of weak classifier number in Adaboost.

After determining the SVM parameters, the number of Adaboost weak classifiers was optimized. As shown in Figure 17, the model’s accuracy reaches 98.02% when the number of weak classifiers is 14. Further increases in the number of classifiers yield limited improvements, and an excessive number of classifiers may waste computational resources. Therefore, the number of weak classifiers is set to 14 during training.

#### 5.4.5. Performance Evaluation and Comparison of Classification Models

To evaluate the effectiveness of the proposed method for fault diagnosis, different classification models are employed based on the input data type. For two-dimensional images, VGG-16 and ResNet CNN models are used [39,40], while an Adaboost-SVM model is applied to image feature vectors extracted using the ORB algorithm. The fault diagnosis dataset includes both field-measured and simulated data, with fault types serving as classification labels. Various signal-to-image conversion methods generate feature maps or extract feature vectors, and models are trained using optimized parameters. The validation set is used for hyperparameter tuning, and model performance is assessed on the test set.

**Figure 18:**
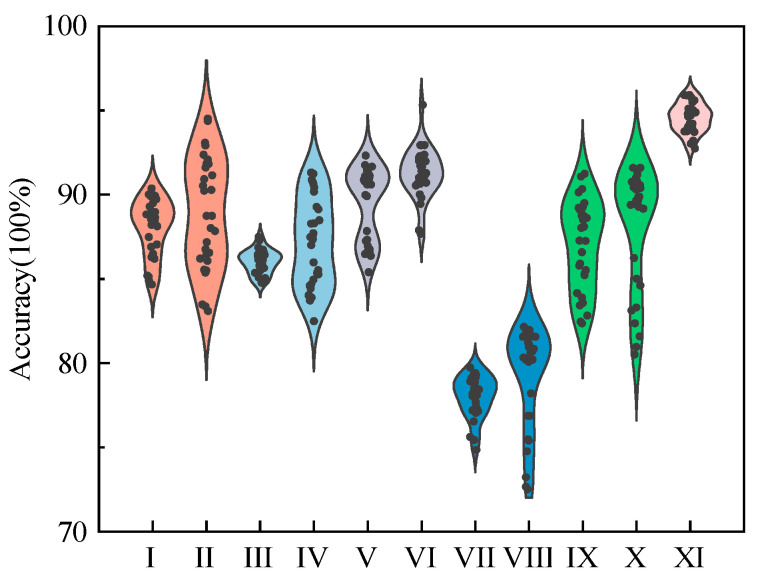
Classification accuracy of different signal-to-image transformation methods.

Classification performance is evaluated using accuracy, the ratio of correctly classified samples to total samples. Each model is tested 30 times, and the average accuracy is reported. Since methods other than ISDP cannot jointly model three-axis vibration signals, each axis’s data is processed separately, and the highest accuracy among the three axes is used for comparison. The results are summarized in Table 7 and Figure 18.

As shown in Table 7 and Figure 18, the ISDP + ORB + Adaboost-SVM method outperforms all other methods in fault recognition accuracy, training efficiency, and testing time. After 30 trials, the method achieved 94.00% accuracy, with a training time of 60 s and a testing time of 8.30 s. Compared to deep learning models like ISDP + ResNet (91.50% accuracy, 5980 s training time), the proposed method is significantly faster and more practical for real-time applications. In terms of model stability, the proposed method exhibits less variation in accuracy across trials, demonstrating greater robustness.

To further validate the performance of the proposed Adaboost-SVM model, it was trained and tested using the processed feature vector data. A comparative analysis was conducted on the classification performance of five machine learning models: SVM, LightGBM, Adaboost-BP, XGBoost-SVM, and CNN. Table 8 presents the average classification performance of each model after 30 repeated trials.

By evaluating various indicators, the Adaboost-SVM model achieved 94.00% $\bar{\mathrm{Pr}e}$ and an $\bar{F1}$ score of 93.50%, outperforming other models in recognizing complex fault modes. Adaboost-SVM also demonstrated superior computational efficiency, with a training time of 60.00 s and a testing time of 8.30 s, significantly faster than Adaboost-BP (120.42 s training, 9.45 s testing) and CNN (160.97 s training, 9.96 s testing). By combining Adaboost ensemble learning with the classification power of SVM, Adaboost-SVM enhances model stability, prevents overfitting, and maintains high accuracy. In contrast to CNN, which is computationally expensive, Adaboost-SVM’s speed makes it ideal for real-time applications with stringent efficiency requirements. While models like SVM and LightGBM are computationally efficient, their accuracy is generally lower, particularly for multi-category fault diagnosis. Overall, Adaboost-SVM’s outstanding performance in accuracy, efficiency, and stability makes it highly suitable for on-site deployment and fault diagnosis in resource-constrained environments.

## 6. Conclusions

This study addresses key challenges in the fault diagnosis of excitation dry-type transformers, including the underutilization of multi-axis vibration data, inefficient feature extraction, and limited recognition accuracy under complex conditions. To overcome these challenges, a multi-channel visual feature fusion method is proposed, integrating an improved ISDP with PSO-based parameter optimization and ORB feature extraction within a lightweight Adaboost-SVM framework. This approach preserves time-frequency features through three-axis feature aggregation, optimizes parameters for better feature discrimination, and utilizes ORB for efficient local detail extraction, reducing computational overhead while maintaining high accuracy. Its effectiveness is validated through simulations and field data under various fault conditions. The main conclusions of this study are as follows:

- Enhanced Feature Representation: The three-axis aggregation significantly improves feature representation. The improved ISDP fuses time- and frequency-domain data from all three axes. With PSO, this method outperforms traditional techniques like SDP, GAF, and MTF in terms of inter-class separability and intra-class consistency;
- Improved Computational Efficiency via ORB: ORB feature extraction enhances efficiency and sensitivity to local details, capturing distortions while reducing redundant dimensions, all while maintaining feature integrity;
- Superior Adaboost-SVM Performance: The Adaboost-SVM model achieves 94.00% accuracy, with a training time of 60 s and a testing time of 8.30 s. It outperforms models like CNN, SVM, and LightGBM, making it highly effective for real-time fault diagnosis;
- Stability and Adaptability: The method demonstrates strong stability and adaptability in multi-class fault diagnosis, maintaining minimal variation across different fault types and conditions. It is effective in resource-constrained environments, well-suited for real-time monitoring, and provides valuable support for operational decision-making, with potential for broader application in other transformer types.
- It is worth noting that although this study examined various operating conditions and parameter variations, the focus was primarily on single-excitation dry-type transformers. Cross-validation with devices of different capacities and core geometries has not yet been conducted but will be addressed in future research. However, the proposed diagnostic framework is model-independent and based on the vibration characteristics of the general electromechanical mechanism, suggesting that it is transferable across different transformer types.

## Figures and Tables

**Table 1:** Overview of transformer fault diagnosis methods.

Key Methods	Core Principles	Advantages	Disadvantages	Application Fields
Dissolved Gas Analysis (DGA) [5,6]	Detects gas types and concentrations in transformer oil to identify faults.	Mature technology; rich data accumulation.	Slow response; hard for real-time diagnosis.	Fault diagnosis of oil-immersed transformers.
Traditional Electrical Detection [7]	Measures parameters (e.g., resistance) to detect faults.	Simple, low-cost, versatile equipment.	Requires shutdown; low accuracy; human-dependent.	Fault screening for small to medium-sized transformers.
Partial Discharge Detection [8,9]	Captures discharge signals to locate faults.	Early fault detection; strong fault location.	Susceptible to interference; complex equipment.	Online monitoring of transformer insulation faults.
Single Vibration Signal Analysis [10,11]	Analyzes vibration signals for mechanical faults.	Non-invasive; sensitive to mechanical faults.	Prone to noise; limited by single-channel data.	Initial mechanical fault detection in transformers.
Deep Learning Diagnostics [12]	Classifies faults using deep learning (e.g., CNN, BP neural networks).	High accuracy; automatic feature extraction.	Needs labeled data; high computational cost.	Offline diagnosis of labeled transformer data.
Multi-source Signal Fusion [13]	Combines vibration, noise, and electrical signals for improved diagnosis.	Comprehensive; enhances accuracy and robustness.	Requires synchronized data and complex algorithms.	Multi-fault diagnosis under complex conditions.

**Table 2:** Main parameters of the excitation dry-type transformer.

Geometric Parameters	Value	Technical Parameters	Value
Low Voltage Winding Inner/Outer Radius	278/303 mm	Number of Phases	3
High Voltage Winding Inner/Outer Radius	385/415 mm	Rated Voltage (High/Low Voltage Windings)	24/1.1 kV
High/Low Voltage Winding Height	1020 mm	Rated Current (High/Low Voltage Windings)	231/5039 A
Core Radius	475 mm	Rated Capacity	3 × 3200 kVA
Core Height	2070 mm	Rated Frequency	50 Hz
Silicon Steel Sheet Thickness	3.3 mm	Insulation Class	F

**Table 3:** Elastic modulus parameters for different degrees of loosening.

Parameter	Normal	20% Loosening	40% Loosening
Elastic Modulus (Pa)	1.16 × 10^11^	9.28 × 10^10^	6.96 × 10^10^

**Table 4:** Parameterized scanning parameter settings.

Parameter	Initial Value	Final Value	Step Size
High-voltage Side Voltage	23.5 kV	29.5 kV	0.1 kV
High-voltage Side Winding Resistance	0.5 Ω	5 Ω	0.1 Ω
Low-voltage Side Winding Resistance	0.01 Ω	0.3 Ω	0.002 Ω
Low-voltage Side Load Resistance	10 Ω	100 Ω	1 Ω

**Table 5:** Dataset description.

Operating Condition	U_N_ (No-Load)	90%U_N_ (No-Load)	80%U_N_ (No-Load)	U_N_ (β = 75%)	U_N_ (β = 80%)	Total
Normal Operation	120/40/40	120/40/40	120/40/40	120/40/40	120/40/40	600/200/200
Winding Loosening	120/40/40	120/40/40	120/40/40	120/40/40	120/40/40	600/200/200
Core Loosening	120/40/40	120/40/40	120/40/40	120/40/40	120/40/40	600/200/200
Winding Eccentricity	120/40/40 *	120/40/40 *	120/40/40 *	120/40/40 *	120/40/40 *	600/200/200

* Indicates that the data are from simulation results.

**Table 6:** Signal similarity evaluation results.

Operating Condition	DTW Distance	Spectral Coherence
No-load Normal Operation	0.23	0.76
No-load Winding Loosening	0.39	0.83
No-load Core Loosening	0.37	0.78
75% Load Normal Operation	0.35	0.83

**Table 7:** Performance comparison of different signal-to-image transformation methods.

Serial Number	Method	Accuracy (%)	Training Time (s)	Testing Time (s)
Ⅰ	GAF + VGG-16	87.50	6200.00	38.00
Ⅱ	GAF + ResNet	87.90	7500.00	40.80
Ⅲ	SDP + VGG-16	85.50	5500.00	18.50
Ⅳ	SDP + ResNet	86.50	5710.00	22.80
Ⅴ	ISDP + VGG-16	89.00	5600.00	22.80
Ⅵ	ISDP + ResNet	91.50	5980.00	26.10
Ⅶ	RP + VGG-16	77.50	5700.00	24.40
Ⅷ	RP + ResNet	79.00	6030.00	27.20
Ⅸ	MTF + VGG-16	86.50	6800.00	32.00
Ⅹ	MTF + ResNet	88.00	6900.00	37.10
XI	ISDP + ORB + Adaboost-SVM	94.00	60.00	8.30

**Table 8:** Comparative analysis of classification performance across models.

Model	$\bar{\mathrm{Pr}e}$	$\bar{F1}$	*t_d_*	*t_s_*
SVM	85.53%	84.20%	51.03 s	6.95 s
LightGBM	91.25%	89.71%	65.02 s	7.81 s
Adaboost-BP	93.12%	92.72%	120.42 s	9.45 s
XGBoost-SVM	92.59%	91.21%	64.93 s	7.89 s
CNN	90.70%	89.74%	160.97 s	9.96 s
Adaboost-SVM	94.00%	93.50%	60.00 s	8.30 s

## Data Availability

The data used in the analysis presented in the paper will be made available, subject to the approval of the data owner.

## Abbreviations

The following abbreviations are used in this manuscript:

SDP	Symmetrized Dot Pattern
ISDP	Improved Symmetrized Dot Pattern
PSO	Particle Swarm Optimization
ORB	Oriented FAST and Rotated BRIEF
Adaboost-SVM	Adaptive Boosting Support Vector Machine
SVM	Support Vector Machine
GAF	Gramian Angular Field
RP	Recurrence Plot
MTF	Markov Transition Field
CNN	Convolutional Neural Network
FFT	Fast Fourier Transform
BP	Backpropagation
VFRA	Vibration Frequency Response Analysis
GASF	Gramian Angular Summation Field
RBF	Radial Basis Function
DAG	Directed Acyclic Graph
DTW	Dynamic Time Warping
LightGBM	Light Gradient Boosting Machine
Adaboost-BP	Adaboost with Backpropagation
XGBoost-SVM	Extreme Gradient Boosting with Support Vector Machine
